# Bringing Antonovsky's salutogenic theory to life: A qualitative inquiry into the experiences of young people with congenital heart disease

**DOI:** 10.3402/qhw.v11.29346

**Published:** 2016-03-01

**Authors:** Silke Apers, Jessica Rassart, Koen Luyckx, Leen Oris, Eva Goossens, Werner Budts, Philip Moons, for the I-DETACH Investigators

**Affiliations:** 1 Department of Public Health and Primary Care, KU Leuven—University of Leuven, Leuven, Belgium; 2 School Psychology and Child and Adolescent Development, KU Leuven—University of Leuven, Leuven, Belgium; 3 Research Foundation Flanders (FWO), Brussels, Belgium; 4 Division of Congenital and Structural Cardiology, University Hospitals Leuven, KU Leuven—University of Leuven, Leuven, Belgium; 5 Institute of Health and Care Sciences, Gothenburg University, Gothenburg, Sweden

**Keywords:** Chronic disease, heart defects congenital, salutogenesis, sense of coherence, qualitative research

## Abstract

**Objective:**

Antonovsky coined sense of coherence (SOC) as the central concept of his salutogenic theory focusing on the origins of well-being. SOC captures the degree to which one perceives the world as comprehensible, manageable, and meaningful. Life events and resources are considered to be the building blocks of a person's SOC. However, mainly quantitative studies have looked into the role of life events and resources. Therefore, the present study aims to gain a deeper insight into the experiences of patients with congenital heart disease (CHD) regarding resources and life events.

**Method:**

For this qualitative study, patients were selected from the sample of a preceding study on development of SOC (*n* = 429). In total, 12 young individuals with CHD who had either a weak (*n* = 6) or strong SOC (*n* = 6) over time were interviewed (8 women, median age of 20 years). Data analysis was based on the constant comparative method as detailed in the Qualitative Analysis Guide of Leuven. Commonalities and differences between patients from both groups were explored.

**Results:**

The following themes emerged: (1) self-concept; (2) social environment; (3) daytime activities; (4) life events and disease-related turning points; (5) stress and coping; and (6) illness integration. Additionally, the degree of personal control was identified as an overarching topic that transcended the other themes when comparing both groups of patients.

**Conclusion:**

These results may have implications for the structure and content of interventions improving well-being in young people with CHD.

Why do some people thrive in the face of adversity, whereas others succumb to it? To contextualize this observation, Antonovsky provided a theoretical framework called the salutogenic theory with sense of coherence (SOC) as the central construct (Antonovsky, 1987). The salutogenic theory focuses on the origins of health determined by the strength of a person's SOC. Figure 1 provides a simplified outline of the different elements of the salutogenic theory. SOC portrays the degree to which a person perceives the world and inevitable stressful events encountered in life as comprehensible, manageable, and meaningful. The strength of a person's SOC is shaped by negative or positive life events and internal (e.g., personality) or external resources (e.g., social support). In turn, a strong SOC facilitates adaptive coping in stressful situations leading to a favorable health outcome (Fig. 1) (Antonovsky, 1987). SOC is a globally relevant concept for which a cross-culturally applicable instrument is available (i.e., the Orientation to Life questionnaire) (Eriksson & Lindström, 2005). The importance of SOC in optimizing (young) people's health and well-being has been examined in countries throughout the world (Eriksson & Lindström, 2005; Rivera, García-Moya, Moreno, & Ramos, 2013). More specifically, previous studies have confirmed the relationship between SOC and both patient-reported and clinical outcomes, such as a satisfying quality of life and reduced mortality (Eriksson & Lindström, 2007; Surtees, Wainwright, Luben, Khaw, & Day, 2003). Hence, SOC has been recognized as an internationally meaningful concept to focus on in patients with chronic conditions (Delgado, 2007).

**Figure 1:**
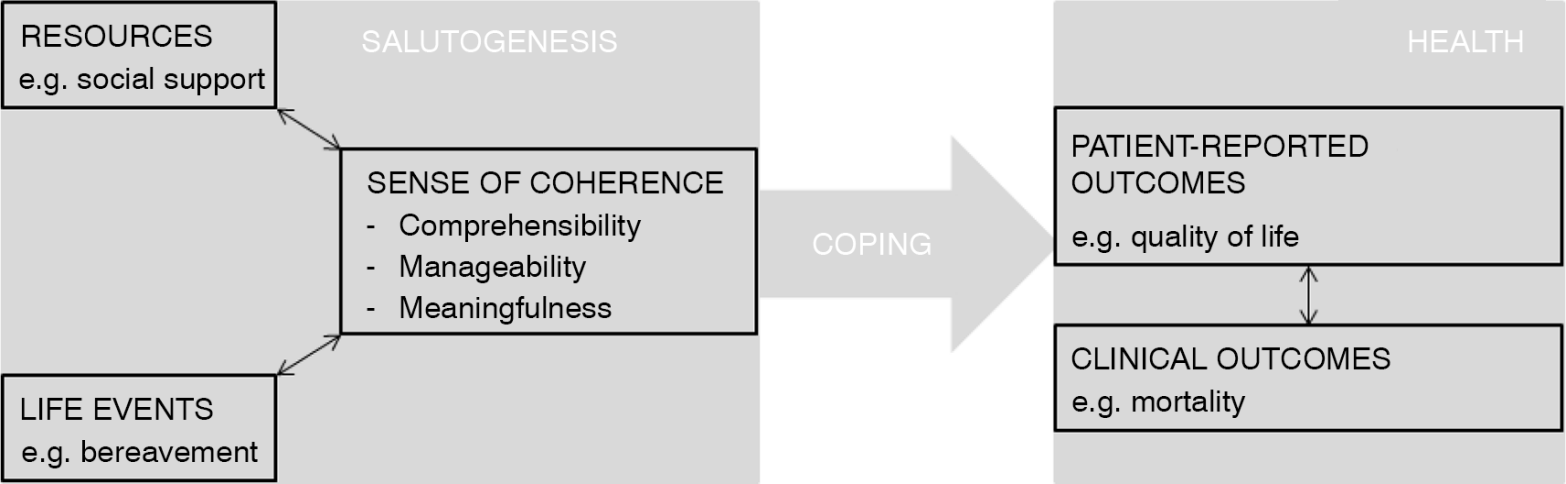
Simplified outline of the different elements in the salutogenic theory.

Regarding the development of a person's SOC, adolescence and early adulthood are deemed to be crucial phases. In this context, patients with congenital heart disease (CHD) can serve as an excellent sample case to study SOC, as CHD is a life-cycle disease (Marelli, 2014) which implies that it can influence the development of SOC from an early age on. CHD is the most common type of birth defect (Van der Linde et al., 2011) and can be defined as structural abnormalities of the heart and/or intrathoracic great vessels that are actually or potentially of functional significance (Mitchell, Korones, & Berendes, 1971). A preceding longitudinal study performed by our research group examined the development of SOC in a sample of young people with CHD over a 27-month period (Apers et al., 2015). Four substantially different subgroups of SOC development were identified, including a group of patients with a *Consistently High*, *Intermediate Stable*, *Intermediate Decreasing*, and *Chronically Low* SOC. Hence, patients varied in the strength of their SOC over time and patients with a weak SOC appeared to suffer most from depressive symptoms, loneliness, a lower quality of life, and a lower level of perceived health (Apers et al., 2015).

Life events and resources are considered to be the building blocks of a person's SOC. However, to date, mainly quantitative studies have looked into the role of life events and resources, suggesting, for example, that family context is the most influential factor in terms of SOC development (García-Moya, Moreno, & Jiménez-Iglesias, 2013) and that negative life events lower a person's SOC (Volanen, Suominen, Lahelma, Koskenvuo, & Silventoinen, 2007). However, little in-depth information on these SOC-shaping factors is currently available (Feldt et al., 2011; Hakanen, Feldt, & Leskinen, 2007). Because the concept of SOC is subjective, complex, and context-bound, qualitative research can help to deepen the current understanding of the role of life events and resources. Such knowledge can guide health professionals in strengthening existing resources or making resources available for young people with a chronic condition, such as CHD. A detailed assessment of the direct perspective of the patient with CHD is needed to provide greater insight into this phenomenon. Therefore, this study aims to gain insight into the experiences of young patients with CHD regarding resources and life events. In doing so, we aim to provide valuable information to promote health and well-being in this patient group, and potentially in the broader field of chronic disease, guided by salutogenic theory.

## Methods

A qualitative, explorative design was chosen to generate a deeper understanding of the experiences of young people with CHD within the context of Antonovsky's salutogenic theory (Antonovsky, 1987), which we used as a conceptual framework for purposive sampling and data analysis (Bowen, 2006).

### Study population

**Figure 2:**
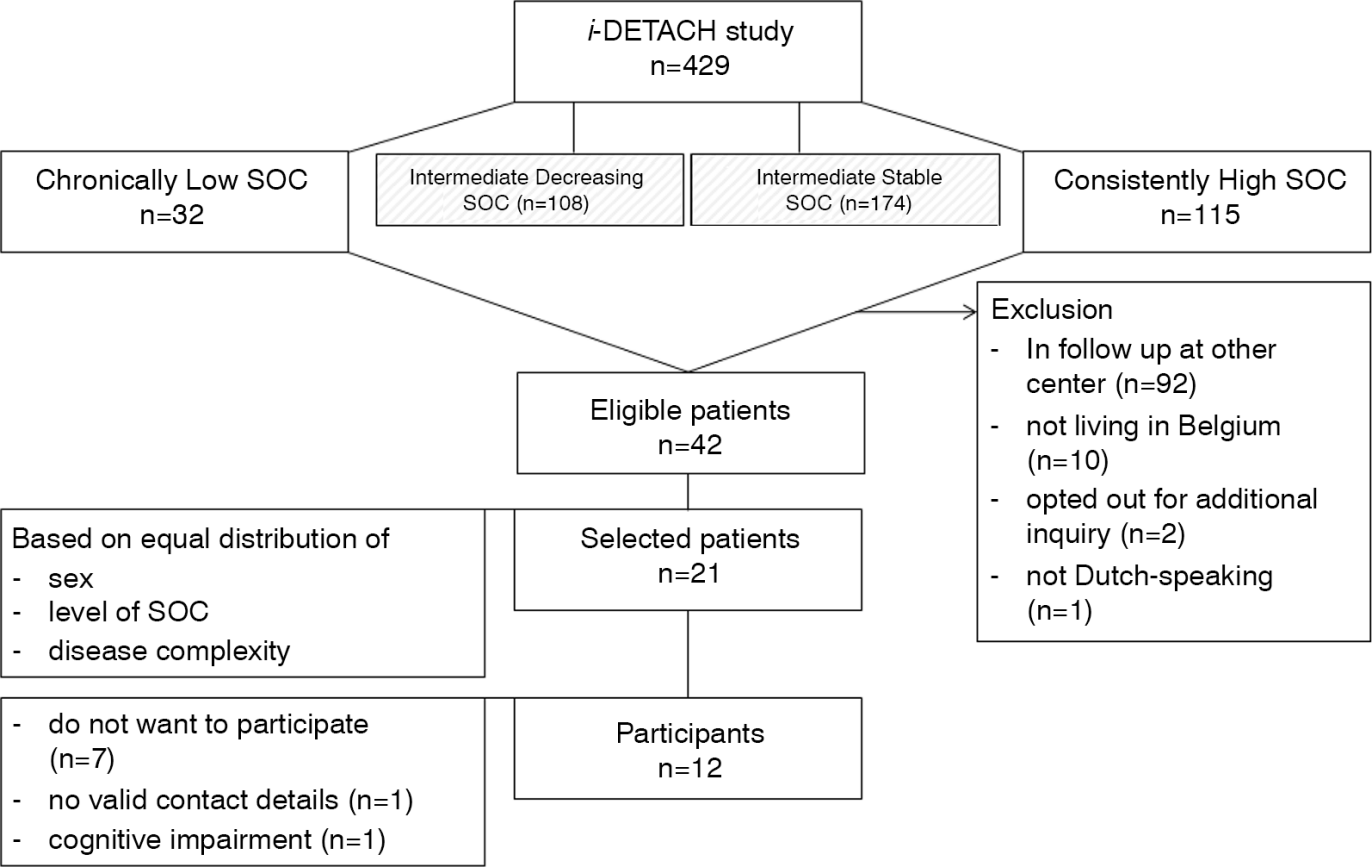
Sample selection. SOC: sense of coherence.

We used a three-step procedure to purposefully select a sample of participants from the quantitative *i*-DETACH project (Information technology Devices and Education program for Transitioning Adolescents with Congenital Heart disease). First, patients with a weak and strong SOC over time (i.e., *Chronically Low* subgroup, *n=*32; *Consistently High* subgroup, *n* = 115) were identified in a previous longitudinal study (Apers et al., 2015). These patients with marked scores on SOC were selected because they were believed to be information-rich cases from whom we can learn most (Patton, 2001). Second, 105 of the 147 selected patients were excluded because they were in follow-up at other centers (*n=*92), did not live in Belgium (*n=*10), opted out for additional inquiry after the quantitative study (*n=*2), or did not speak Dutch (*n=*1). Third, we selected a subsample of 21 patients based on an equal distribution in terms of sex, level of SOC, and CHD complexity to ensure a varied sample. These 21 patients received an invitation letter with information about the study. Ten days later, patients were phoned by the interviewer (SA) and scheduled for an interview. Seven patients declined to participate (e.g., too busy); we did not have a valid phone number for one patient; and one patient had to be excluded because of cognitive impairments. As such, 12 patients who had either a weak (*n=*6) or strong (*n=*6) SOC were included in this study. Figure 2 provides an overview of the sampling procedure.

Participants’ characteristics are presented in Table I. Eight women and four men participated, with an age ranging from 18 to 21 years. Participants’ represented all levels of CHD complexity (i.e., simple, moderate, and complex CHD).

**Table I:** Demographic and medical characteristics of young people with congenital heart disease.

Variables	Total group (*n*=12)	Chronically low SOC subgroup (*n*=6)	Consistently high SOC subgroup (*n*=6)
Median age (min–max)	20 years (18–21)	19.5 years (18–21)	20 years (19–21)
Sex			
Women	8	5	3
Men	4	1	3
Student	9	5	4
Employed	3	1	2
Family structure			
Parents who are living together	10	5	5
Parents who are divorced	2	1	1
Disease complexity			
Simple	3	2	1
Moderate	4	2	2
Complex	5	2	3
Cardiac surgery/intervention performed in the past	6	3	3
Self-reported medical conditions	3	2	1

SOC: sense of coherence. Disease complexity was based on Task Force one of the 32nd Bethesda conference.

### Data collection

Twelve face-to-face interviews were conducted from March to April 2013. Depending on patients’ preferences, interviews were conducted in the hospital (*n=*1), their home (*n=*7), or student apartment (*n=*4). The first author (RM, MSc, PhD student) conducted all interviews and had no (professional) relationship with participants. An observer (RN, master's student) was present throughout the interviews to take notes. The interviewer was blinded for participants’ respective level of SOC during all interviews to avoid bias. Interviews were guided by a limited number of open-ended questions focusing on resources and life events by asking how participants respond to and deal with challenges in life (both related and unrelated to their CHD), for example “Can you tell me about a situation or a moment that was difficult for you; can you describe what helped you to deal with it?” Depending on the course of the interview, additional questions or exemplifications were asked. Interviews lasted from 30 to 80 min (58 min on average), were audiotaped, and transcribed verbatim. Two participants requested the interview transcripts but did not provide any comments or corrections.

The study protocol conforms to the ethical guidelines of the 1975 Declaration of Helsinki and was approved by the University Hospital's Institutional Review Board. All participants were provided with oral and written information about the aim of the study. Written informed consent was obtained from all participants.

### Data analysis

The process of data analysis was based on the Qualitative Analysis Guide of Leuven (QUAGOL) which divides data analysis into two major phases subdivided into 10 steps (Dierckx de Casterlé, Gastmans, Bryon, & Denier, 2012). The analysis process was a team activity and performed using the constant comparative method. This is a comprehensive and systematic method in which researchers are constantly moving between the different steps of the analysis process. The first analytic phase included five steps of preparation of the coding process: (1) thorough (re)reading of interviews to familiarize with the data and to get a holistic understanding of each interview; (2) writing a narrative report that summarized the key storylines to capture the essence of each interview; (3) creating a conceptual interview scheme from each interview in which participants’ experiences were translated into concepts; (4) fitting test and adaptation of the conceptual interview schemes by rereading the interviews as a first backward-forward moment; and (5) the constant comparison process in which common themes were identified by comparing all conceptual interview schemes to create one overarching scheme. The second phase of the data analysis comprised five steps of the actual coding process: (6) creating a common list of concepts which was then introduced into NVivo 10^®^ without introducing hierarchy or links between concepts yet; (7) coding of the interviews with the list of concepts while critically reviewing and optimizing the coding list; (8) analyzing and defining the concepts through across-case analysis (i.e., reviewing all citations linked to a concept); (9) extracting the essential structure by integrating all concepts in one story line in response to the research question followed by verification of the framework against all interviews; and (10) describing the results by reconstructing the story of the patients including a final rereading of the interviews and peer-debriefing. All researchers involved in this study were blinded for participants’ respective level of SOC during data collection and most of the analysis process (i.e., until across-case analysis). All interviews were analyzed by the interviewer (SA) and the second author (MSc, PhD student). Data analysis and emergent results were continuously discussed within the research team (i.e., interdisciplinary triangulation) (Kimchi, Polivka, & Stevenson, 1991) until consensus was reached. The coding was carried out in a manner that was open to what participants had to say and stayed semantically close to their wording.

After identifying common themes in the data, results from patients with a weak and strong SOC were compared and, hence, their level of SOC was disclosed to the authors at this stage of the analysis process. Commonalities and differences between patients from both groups were explored. To elicit the level of data saturation (i.e., the point at which no new insights emerged) we developed a saturation grid (Table II) (Morse, 1995). This grid summarizes which of the identified (sub)themes were covered in each interview. No new (sub)themes were mentioned by patients in the final interviews and all themes had broad coverage across the interviews. Hence, saturation was reached for this study (Table II).

**Table II:** Data saturation grid.

	Interview number											
	
Theme	1*	2	3*	4	5*	6*	7*	8*	9	10	11	12
Self-concept												
Personality	X	X	X	X	X	X	X	X	X	X	X	X
Identity		X	X	X		X		X	X	X	X	X
Self-worth	X		X	X	X	X	X	X	X	X	X	X
Social environment												
Family of origin	X	X	X	X	X	X	X	X	X	X	X	X
Peer contacts	X	X	X	X	X	X	X	X	X	X	X	X
Partner/in-laws	X		X	X				X		X		X
Daytime activities												
Study	X	X	X	X	X	X	X	X	X		X	X
Work	X	X								X		
Leisure activities	X	X	X	X	X	X	X	X	X	X	X	X
Life events and turning points	X	X	X	X	X		X	X	X	X		X
Negative life events	X	X	X	X	X		X	X	X	X		X
CHD-related turning points	X	X	X	X	X					X		
Stress and coping												
Stress	X	X		X	X	X	X	X	X	X	X	X
Adaptive coping	X	X	X	X	X	X	X	X	X	X	X	X
Maladaptive coping	X		X	X	X	X	X	X	X	X	X	X
Illness integration												
Impact of illness on daily life	X	X	X	X	X	X	X	X	X	X	X	X
Personal growth				X					X	X		
Understanding of illness	X	X	X	X	X	X	X	X	X	X	X	X
Degree of personal control	X	X	X	X	X	X	X	X	X	X	X	X

CHD: congenital heart disease.

* Patients with a weak sense of coherence.

## Results

Six common intertwined themes were identified: (1) self-concept; (2) social environment; (3) daytime activities; (4) life events and disease-related turning points; (5) stress and coping; and (6) illness integration. In addition, the degree of personal control emerged as an overarching topic that transcended the six common themes when comparing patients with a weak and strong SOC. Exemplifying interview quotes for each (sub)theme are presented in Supplementary Table I.

### Self-concept

Patients talked about how they see themselves in terms of their personality (i.e., acting in line with broad personality traits and social roles), identity (i.e., making motivated choices, planning your life, and complying with certain goals), and self-worth (i.e., extent to which goals are being achieved and expectations fulfilled), representing different layers of their self-concept (McAdams, 2013).

Patients with CHD from both groups shared some characteristics of their *personality*, such as being stubborn and emotional. Patients with a weak SOC generally described themselves as shy or withdrawn, worried, ruminative, insecure, and having difficulties with managing stress. In contrast, patients with a strong SOC talked about being good in putting things into perspective and having a positive mindset (Supplementary Table I).

Patients discussed their *identity* in terms of having an idea about their future. Patients with a strong SOC typically had a more pronounced idea about their future, including ideas concerning their study or family planning. They talked about making deliberate choices that fit their interests after thorough exploration, whereas patients with a weak SOC explored less and sometimes only had a hazy picture of their future (Supplementary Table I).

Patients from both groups considered having a good social environment to be very important for their *self-worth*. However, patients with a weak SOC talked about not accepting themselves in the past or only accepting themselves because they felt they could not change who they are. Moreover, they spoke about experiences damaging their self-worth (e.g., negative feedback at school). Conversely, patients with a strong SOC more often made a clear statement about accepting who they are and being happy about themselves in general and regarding their studies, social relationships, and appearances (Supplementary Table I).

### Social environment

Patients’ social environment could be subdivided into their family of origin, peer contacts, and their partner/in-laws. This social environment has been referred to as a person's microsystem (i.e., settings or contexts in which the individual interacts as a direct participant) and is known to have a crucial influence on personal development (Huebner, 2009).

Regarding their *family of origin*, for patients in both groups, their mother played an essential role in providing emotional support, whereas their father appeared to have a less prominent role that mainly focused on providing study-related support. Patients from both groups talked about how their family of origin plays a significant role in who they are as a person and that they were lucky to grow up in a supportive family environment, including their siblings and grandparents. Patients with a strong SOC mentioned a broader family support network, including their cousins and aunts. Interestingly, patients with a weak SOC attached more importance to their parents, whereas patients with a strong SOC also greatly valued the support from their friends or partner (Supplementary Table I).

Indeed, patients’ *peer contacts* played an important role in determining their outlook on life and their self-worth. However, patients with a weak SOC talked about negative experiences with their social environment, such as being bullied by their classmates. Additionally, patients with a weak SOC generally felt that their friends could not fully understand their medical situation, whereas patients with a strong SOC perceived their friends as an important source of support regarding their CHD (Supplementary Table I).

Finally, patients from both groups felt their *partner* was an important source of support, although some patients with a weak SOC experienced a lack of support from their partner regarding their CHD (Supplementary Table I).

### Daytime activities

This theme covers aspects of patients’ study, work, and leisure activities, all being important domains of exploration to young people (Arnett, 2007).

Patients from both groups indicated that they have made a good *study* choice. Their study played an important role in their lives and study results influenced their self-worth. However, they also talked about their study as a source of stress, such as experiencing failures at school. Patients from both groups differed as young people with a weak SOC more often expressed negative study-related experiences (e.g., weak study results) which made them feel disappointed in themselves and fed their insecurity. In contrast, if patients with a strong SOC talked about failing at their study, they talked about finding a way out with the help from their parents, such as choosing a new study that better fits their interests and competencies (Supplementary Table I).

Patients who were working expressed that their *work* was a major source of satisfaction to them (Supplementary Table I).

Finally, patients mentioned a multitude of *leisure activities* which are a source of fulfillment and relaxation. Patients with a weak SOC were less involved in group leisure activities, whereas leisure activities were an important way of having social contacts for patients with a strong SOC (Supplementary Table I).

### Life events and disease-related turning points

Patients talked about negative life events and disease-related experiences that proved to be a turning point. Such key critical events are known to influence adjustment to chronic illness (Moss-Morris, 2013).

Patients from both groups experienced negative *life events*, including divorce of parents, school problems, and bereavement. It became apparent that patients with a weak and strong SOC differed in the meaning they attached to these negative events and, consequently, the outcome of the event. Patients with a strong SOC reappraised negative life events by identifying positive aspects. They considered these stressful circumstances as valuable learning experiences that motivated them, reinforced the use of certain coping strategies, and, hence, led to personal development. In contrast, patients with a weak SOC did not resolve negative life events and mainly expressed their emotional responses to these events, such as feelings of impaired self-worth (Supplementary Table I).

In addition, patients talked about *disease-related turning points*, such as having difficulties during physical activity that made them realize the impact of their condition. Patients with a strong SOC spoke about how this helped them accept their condition. For example, one patient explained how, during a conversation with her cousin, she came to realize that her scar was the reason that she was still alive which gave her a feeling of survivorship and gratitude. On the other hand, patients with a weak SOC talked about how these moments made them realize their limitations and expressed their negative feelings, such as never being carefree again (Supplementary Table I).

### Stress and coping

Patients talked about experiencing stress (i.e., appraisal of the environment as exceeding their resources) and their strategies to cope with this (i.e., efforts to manage demands) (Lazarus & Folkman, 1984).

Patients from both groups experienced *stress* related to school. However, patients with a strong SOC did not experience a lot of stress, while patients with a weak SOC were easily stressed out and felt they were not good in dealing with stress (Supplementary Table I). For the latter group, interpersonal stressors played a significant role, such as arguing with their parents.

Patients used both adaptive and maladaptive *coping* strategies. For example, both groups of patients sought professional support for their problems (i.e., adaptive coping), such as seeing a psychologist. In terms of maladaptive coping, patients sometimes made use of passive coping strategies (e.g., resigning themselves to the situation). Patients with a strong SOC made more use of adaptive coping strategies (e.g., social support seeking), whereas patients with a weak SOC applied more maladaptive coping strategies (e.g., rumination). For instance, patients with a strong SOC actively looked for solutions to their problems through seeking emotional social support (e.g., talking to their friends). Conversely, patients with a weak SOC seemed to worry a lot about how they would cope with certain demands and were often occupied by negative thoughts (Supplementary Table I).

### Illness integration

This theme covers three aspects of illness integration (i.e., integrating the illness into one's identity) (Leventhal, Idler, & Leventhal, 1999): the impact of illness on patients’ daily life (i.e., physical and emotional/social impact); the experience of personal growth (i.e., benefit finding or identifying positive ways in which their lives have changed as a result of a stressor) (Helgeson, Reynolds, & Tomich, 2006); and patients’ understanding of their illness.

Compared to patients with a weak SOC, the *impact of illness on the daily life* of patients with a strong SOC was greater. First, they experienced more physical restraints due to their CHD (e.g., not being able to bike to school). This required patients to adapt themselves to the situation at hand (e.g., choosing other, less demanding leisure activities). In contrast, patients with a weak SOC mostly experienced minor physical restraints, such as feeling tired from time to time (Supplementary Table I). Second, in terms of the emotional and social impact of their CHD, patients with a strong SOC had mixed ideas about whether their illness had made them stronger. They did agree about the fact that having CHD positively influenced their outlook on life; it made them more perseverant and helped them to put things into perspective. For patients with a weak SOC, sorrows and worries about their heart played the leading part, although they felt their CHD had almost no impact on their life. In other words, patients with a weak SOC had more difficulties accepting their illness compared to patients with a strong SOC.

A positive sense of *personal growth* emerged from the stories of some patients with a strong SOC. These patients talked about how they felt enriched by their heart disease and how they were grateful that they were still alive. Nevertheless, they also had negative feelings related to their CHD, such as regretting that they were not able to physically compete with others. However, they did not perceive this impact of CHD as a limitation of their functioning (Supplementary Table I). This sense of personal growth did not emerge from the stories of patients with a weak SOC.

*Understanding your illness* is part of accepting and integrating it into your life. Both groups of patients felt they received sufficient information about their CHD from health professionals, which they perceived as a source of support. Nevertheless, patients with a weak SOC did not seem to possess good knowledge about their heart defect (e.g., an incorrect idea about disease progression). Patients with a strong SOC, on the other hand, gave very detailed descriptions of their defect and explained they knew very well about their CHD and its consequences (Supplementary Table I).

### Degree of personal control

Patients’ degree of personal control was the connecting thread between all themes and refers to their beliefs regarding the extent to which they are able to control different aspects in their lives. The importance of personal control to psychosocial functioning has been established by numerous theorists (Skinner, 1996). Personal control is also included in the common sense model (i.e., a theory on illness management) as an illness perception (i.e., perceptions of the person's ability to control the illness) (Dempster, Howell, & McCorry, 2015; Leventhal, Leventhal, & Contrada, 1998).

Patients with a weak SOC appeared to experience a low *degree of personal control* (e.g., not being able to resolve study-related problems), whereas patients with a strong SOC seemed to experience a good sense of personal control (e.g., being allowed by their parents to make their own decisions and to be independent) (Supplementary Table I).

## Discussion

This qualitative study inquired into the experiences of young people with CHD from a salutogenic perspective. Including patients with both a weak and strong SOC made it possible to uncover responses that were consistent across patients and to identify unique reactions in both groups.

Patients from both SOC groups did not differ in terms of going through negative life events, but they did differ in terms of the availability and use of resources to deal with such events. Relatedly, a study by Hochwälder and Forsell (2011) did not produce strong evidence that negative life events lower a person's SOC, which is in line with the fact that patients from both groups in this study had equal experiences with negative life events. These results suggest that the experience of going through a negative life event in itself might not be decisive for patients’ SOC.

Concerning internal resources, a potentially important finding is the differences that patients described regarding their personality. Patients with a weak SOC talked about being insecure, always worried, and having coping difficulties, whereas patients with a strong SOC highlighted their ability to put things into perspective and to think positive. Furthermore, patients also differed in the understanding of their heart defect (i.e., another internal resource), as patients with a weak SOC appeared to be less knowledgeable. In terms of external resources, patients with a strong SOC seemed to have a broader social support network as compared to patients with a weak SOC. The social support that patients with a strong SOC received helped them in coping with their CHD and in resolving, for example, study-related problems. In contrast, patients with a weak SOC talked about experiencing stressful social interaction. Interpersonal stressors especially represent a potential threat to young people's well-being and healthy development (Charbonneau, Mezulis, & Hyde, 2009). The importance of support from patients’ family and friends was also identified as a theme in a previous qualitative study using a salutogenic orientation to describe the experiences of young people with a chronic condition (Aho, Hultsjö, & Hjelm, 2015).

Patients with a strong SOC were able to successfully overcome the challenges they faced, which could be a resource in itself. Indeed, patients with a strong SOC experienced a greater impact of CHD on their daily life which might explain the sense of personal growth or enrichment that some of these patients experienced (Helgeson et al., 2006). In order to feel enriched by something you need to be confronted with significant stressors first (Helgeson et al., 2006). For example, dealing with physical limitations from an early age on stimulated these patients to adapt themselves to other situations (e.g., study-related issues) (Tong et al., 1998). Young patients with a weak SOC did not share experiences of personal growth, but instead talked about repeated (unresolved) negative study-related experiences. Such experiences damaged their self-worth and, thus, could have made them more vulnerable. Apparently, negative life events have the potential to elicit distress as well as to lead to psychological resilience and personal growth in young patients with CHD depending on the availability and use of resources, which is in line with Antonovsky's theory (Antonovsky, 1987). Furthermore, prior qualitative studies identified groups of patients that have a lot of characteristics in common with the group of patients with a strong SOC in the current study. More specifically, Claessens et al. (2005) identified a group of adults with CHD who successfully adapted to and integrated limitations into their lives, was good at putting things into perspective, and felt they were in control. In addition, a recent study by Berghammer, Brink, Rydberg, Dellborg, and Ekman (2015) of young adults with a univentricular heart described that CHD contributed to patients’ outlook on life, they integrated CHD into their life, they were grateful to be alive, they experienced their scar positively, and their CHD even contributed to their personal strength.

Another important finding in this study was the emergence of the overarching topic degree of personal control. Young people with a weak SOC were low in control, which became apparent in how they dealt with negative life events, for example. Patients with a strong SOC managed to turn these events into something positive (i.e., take back control by making their own decisions), whereas patients with a weak SOC gave way to sorrow. Furthermore, the finding that patients with a weak SOC experienced more interpersonal stressors could be related to their lack of control in general as relationship-oriented factors are less likely to be under a person's control (Griffiths, Ryan, & Foster, 2011). For patients with a strong SOC their upbringing appeared to play an important role in their perception of control as their parents encouraged them to be independent. The importance of achieving a sense of control was highlighted in previous studies in patients with CHD as well (Berghammer et al., 2015; Chiang et al., 2015; Cornett & Simms, 2014; Tong et al., 1998). Hence, a sense of control may be an additional resource to deal with negative life events in patients with CHD.

### Data quality and limitations

Trustworthiness was ensured throughout this study (Lincoln & Guba, 1985). First, to guarantee credibility (i.e., congruence of the results with reality), data analysis and emergent results were continuously discussed within the research team. In addition, the analysis method used according to QUAGOL is a comprehensive and systematic method (Dierckx de Casterlé et al., 2012). Second, in terms of dependability (i.e., consistency in the results), the interviews were initially analyzed separately by the first and second author and there was good agreement. Furthermore, use of the COREQ (consolidated criteria for reporting qualitative research) checklist ensured a clear exposition of methods, data collection, and analysis (Tong, Sainsbury, & Craig, 2007). Third, transferability (i.e., applicability to other settings) was assured by reflecting on the results in relation to salutogenic research and qualitative studies in the field of CHD, and by providing a rich description of the results with appropriate quotes. Fourth, confirmability (i.e., objectivity of the results) was attended to by ensuring initial blind analysis.

Nevertheless, bias may arise from conducting interviews with patients having a weak SOC versus patients having a strong SOC as the latter group is more likely to be aware of their emotions and can more easily describe them (Antonovsky, 1987). Furthermore, some patients suffered from a medical condition besides their heart defect which might have influenced their stories as well. As for all qualitative studies, the results must be interpreted in relation to their contexts, time, and place. Overall, all identified (sub)themes were covered broadly, except for partner/in-laws (social environment), work (daytime activities), disease-related turning points, and personal growth (illness integration). In terms of disease-related turning points and personal growth more interviews might be necessary to fully understand and confirm these results.

### Implications

Our results can have implications for interventions aimed at improving health and well-being in young people with a chronic condition worldwide. SOC is a highly actionable concept because of its three components (i.e., comprehensibility, manageability, and meaningfulness) (Antonovsky, 1987). Indeed, studies from different countries have found that SOC can change or be changed over time (Feldt et al., 2011; Hakanen et al., 2007) using different methods, such as patient empowerment, talk-therapy groups, lifestyle intervention programs, or patient education courses (Delbar & Benor, 2001; Fagermoen, Hamilton, & Lerdal, 2015; Forsberg, Björkman, Sandman, & Sandlund, 2010; Langeland et al., 2006).

The first component, comprehensibility, tells us something about how individuals perceive information as structured and predictable (i.e., the problem faced is clear). In this study, patients with a weak SOC were less knowledgeable about their CHD which can compromise their feelings of comprehensibility. Hence, this stresses the importance of providing sufficient and appropriate information about the condition and its implications (Taylor, Gibson, & Franck, 2008). The second component, manageability, tells us something about how individuals perceive that resources are at their disposal to meet demands (i.e., resources to cope with the problem are available). This component is strongly related to a person's sense of control, which clearly differed between both groups of patients to the detriment of patients with a weak SOC. Patient empowerment can be a way of instilling control (Delbar & Benor, 2001). In addition, our results indicate that a perceived lack of resources can be a vital link in the development of a weak SOC highlighting the importance of identifying resources and making them available to patients. One of the most important resources was social support. Hence, health professionals should, for example, involve patients’ family and friends (Taylor et al., 2008). The third component, meaningfulness, says something about how individuals perceive that life makes sense emotionally (i.e., the extent to which one wishes to cope with the problem). In this study, patients with a strong SOC were motivated to cope with negative life events and perceived them as meaningful challenges. Patients with a weak SOC, on the other hand, perceived these events as a burden without attaching a deeper meaning to it. Furthermore, the finding that patients with a weak SOC made more use of maladaptive coping strategies (e.g., passive coping) indicates that these patients tend to give up in advance and sometimes do not make any attempt at making sense of stressors. In healthcare settings, actively involving patients in decision-making is essential to support feelings of meaningfulness (Taylor et al., 2008).

## Conclusion

This qualitative study was guided by Antonovsky's salutogenic theory and aimed to gain insight into the experiences of young patients with CHD regarding life events and resources. We found that patients with a weak and strong SOC did not differ in terms of going through negative life events, but they did differ in terms of the availability and use of resources to deal with these events. As such, our results underscore the importance of internal and external resources in the development and adjustment of patients with a chronic condition such as CHD. These insights can have implications for the content of interventions to improve health and well-being in young people with a chronic condition worldwide.

## Supplementary Material

Bringing Antonovsky's salutogenic theory to life: A qualitative inquiry into the experiences of young people with congenital heart disease
