# Supplementary material for: Bringing Antonovsky's salutogenic theory to life: A qualitative inquiry into the experiences of young people with congenital heart disease
Source: Int J Qual Stud Health Well-being. 2016 Mar 1;11:10.3402/qhw.v11.29346. doi: 10.3402/qhw.v11.29346 (PMC4778384; doi:10.3402/qhw.v11.29346)
Supplement: Bringing Antonovsky's salutogenic theory to life: A qualitative inquiry into the experiences of young people with congenital heart disease [file QHW-11-29346-s001.pdf]

Supplementary Table 1. Quotes supporting the identified themes in young people with congenital heart disease

Themes	Quotes
Self-concept	
Personality	- "*If I do something, I put my heart and soul into it.*" (woman, 19 years old, complex CHD, strong SOC)<br>- "*I've always been shy. Making contact with strangers is a big step for me. But I guess that's just who I am.*" (woman, 21 years old, moderate CHD, weak SOC)
Identity	- *"From Year 4 in Secondary School already, I had clear goals in my life. Teamwork, especially, was top of my list.*" (man, 21 years old, moderate CHD, strong SOC)<br>- "*I'm still unclear about what I want to do after secondary school.*" (woman, 18 years old, moderate CHD, weak SOC)
Self-worth	- "*No, I'm happy with who I am. I have a circle of friends already who like me. And yes, I reckon if you're in with a good crowd, then it's a whole lot easier to accept who you are.*" (man, 20 years old, complex CHD, strong SOC)

		- "*It's gruelling, of course, to have to get back on your feet, time and again. And when you hear them list the issues you still have to address, that makes me more insecure. It makes me uncertain about what I'm able and unable to do.*" (woman, 21 years old, moderate CHD, weak SOC)
Social environment		
	Family of origin	- "*I discussed it with my parents: where do I go from here? So they helped me to consider my options. And we decided that I should redo the whole year. So yes, they have helped me, in particular, to hang on in there.*" (man, 20 years old, complex CHD, strong SOC)<br>- "*The relationship with my brother is, in fact, quite shallow. We're not really close at all. When I was little, my brother always used to say: you must be the milkman's... or you're a bastard child of such and such… as you're not at all like other children. And yes, that's quite hurtful, isn't it, if your own brother says that you're someone else's child.*" (woman, 20 years old, simple CHD, weak SOC)
	Peer contacts	- *"Yes, so me, my nephew and my flatmate – we're all close friends. And we've been holidaying together too, for two years now, a partying holiday to be more precise. In the football team too, I get on well with a couple of other lads and we regularly meet up. To go for a drink or something.*

	I do know a lot of people who are great guys and who I hang out with." (man, 20 years old, complex CHD, strong SOC)
	- "*I had no friends. And if I did have any friends, well, they weren't the right sort of friends. They used me. They knew that if they needed a loan, for instance, that they could borrow off me. But, time and again, that was the last I saw of my money...*" (woman, 20 years old, simple CHD, weak SOC)
Partner/in-laws	- "*That* [partner] *is the main person in my life, right now. We're living together, you see. And yes, if I have any problems, I can always turn to her.*" (man, 20 years old, simple CHD, strong SOC)
	- "*My dear partner – yes, to him I can unload all my problems. What he does, first and foremost, is listen and then often tries to give me good advice.*" (woman, 21 years old, simple CHD, weak SOC)
Daytime activities	
Study	- *"Eventually I ended up in the profession of physiotherapy. That has turned out to be a good choice, I reckon.*" (man, 20 years old, complex CHD, strong SOC)
	- "*That I'm now a qualified nurse. I'm so happy that I made that career choice.*" (woman, 21 years old, simple CHD, weak SOC)

Work	- *"So I'm the person who runs the factory. The job isn't dull though; it offers a lot of variation. And that's great, isn't it? As you learn something new while in the job. You manage and coordinate something, as a team. It follows then, that I have some good workmates.*" (man, 21 years old, moderate CHD, strong SOC)<br>- "*I also work in an old people's home. It's taught me a lot about people and people management.*" (woman, 20 years old, simple CHD, weak SOC)
Leisure activities	- "*I studied karate for a long while at secondary school, and I have still friends that date back from then. It always nice to catch up with them.*" (man, 20 years old, simple CHD, strong SOC)<br>- "*I have currently no hobbies. Dancing, though, was one thing that I did used to do.*" (woman, 21 years old, moderate CHD, weak SOC)
Life events and turning points	
Negative life events	- "*Last year I had long periods of great stress. Although I began in the field of psychology, I decided it wasn't for me and moved to primary education... Were I to go back and do it all again I think I'd do the same as it's taught me a helluva lot... I think that you come out of it stronger. You get to know yourself in whole new way. Where your boundaries lie. So, in the future, I'll be*

	forewarned: ah, I can sense that (same thing) coming… I now have to do this and that..." (woman, 19 years old, moderate CHD, strong SOC)
	- "*I had to drop out of General Secondary Education and go to Technical Secondary Education. At the time, it was a very difficult period for me as, for the first time ever, I had to get to know new people and, worse still, halfway through a school year. Yes, I think I was lucky in that a girlfriend of mine also moved across to Technical Secondary Education. That I at least knew somebody there, as we didn't get a very warm welcome from our new classmates. So yes, that does dent your self-esteem. I felt a bit of a failure for not succeeding in my GSEs and, yes, that makes you more insecure, of course.*" (woman, 21 years old, moderate CHD, weak SOC)
CHD-related turning points	- *"When I fainted I was playing basketball. It came from nowhere, I didn't know what was happening to me. But, from that moment on, it dawned on me: wow, I should have taken things at a more relaxed pace. So that faint, even though I was very young at the time, about ten or so… really brought it home... and* [CHD] *became a key consideration. I repeat, after that faint, my whole perspective changed. I re-prioritized my whole life.*" (man, 21 years old, moderate CDH, strong SOC)

	- “*When I was born the doctors knew that something was wrong with me, but it remained a secret. But then the family doctor, he noticed, at a very tender age, that it really was quite serious. And he took me, on several occasions, to different hospitals, etc. Then when I was about ten years old (a fourth former), it was finally diagnosed as CHD. Before that diagnosis, you could live life and be largely unconcerned about it. It’s not as it’s the first thing you think about when you awake each morning. So it was relatively carefree up till then. That’s still pretty much the case. It’s not a source of great concern, but it does niggle away at the back of your mind.*” (man, 19 years old, complex CHD, weak SOC)
Stress and coping	
Stress	- “*I’m pretty much immune to stress. I don’t know really how to put it – I’m able to handle pressure.*” (woman, 20 years old, complex CHD, strong SOC)<br>- “*There are also bad days when I go to sleep, highly stressed. That I get up the next day and – from the moment I open my eyes – I get a sense of, wow, you’re so on edge. And I stay that way, restless and uneasy.*” (man, 19 years old, complex CHD, weak SOC)
Adaptive coping	- “*I went out and asked many people: what did you do and how did you tackle that? Yes, I talk with many people about different things.*” (woman, 19 years old, moderate CHD, strong SOC)

		- *"And if it all becomes too much, I start to make lists, so that I can tackle things, step by step. That does help me, going through things systematically. Surveying the situation and saying to myself: yes, maybe it'll work if I do it that way.*" (man, 19 years old, complex CHD, weak SOC)
	Maladaptive coping	- "*That you can try to put your mind to other things (besides CHD), but it doesn't last long. I've been down the other path you see, of planning your diary absolutely full of stuff. But then you're run off your feet, you're never at peace, neither your body or soul. And, in the final analysis, you've got no further mentally than where you started. You can't come to terms with CHD like that.*" (woman, 19 years old, moderate CHD, strong SOC)
		- *"At such a moment, you don't know what to do* [negative feedback from a teacher during on-the-job training]. *You're not angry at those people because they wanted to help you but, on the other hand, you think to yourself, damn, they could have told me that earlier. And then you do worry yourself sick about it.*" (woman, 18 years old, moderate CHD, weak SOC)
Illness integration		
	Impact of illness on daily life	- "*I can just about cycle to school, but if I can avoid it, then I will, as I would have to stop a few times en route, and it would be painful.*" (woman, 19 years old, complex CHD, strong SOC)

	- *"I don't have any discomfort any more. It's not as if I have any symptoms of CHD as such.*" (woman, 18 years old, complex CHD, weak SOC)
Personal growth	- "*If I hadn't had that* [i.e., CHD] *than I would have looked at life differently, in any case, and viewed things differently. And then I wouldn't have become the person that I am today and not so mentally tough as I am now. I do think, though, that you're much stronger than if you haven't had that* [i.e., CHD]. *As it changes your outlook on life.*" (woman, 19 years old, moderate CHD, strong SOC)
Understanding of illness	- "*My right ventricle is underdeveloped, to such an extent, that my left ventricle has to do the work of both. Part of the septum between my left- and right ventricles is missing and the positions of my aortic valve and pulmonary artery have reversed. And, in an operation, they detached two hollow veins from my heart and attached them to my aortic valve.*" (woman, 19 years old, complex CHD, strong SOC)
	- *"Then they asked me: which disease do you have? But I couldn't say exactly. I only know that I used to have a tiny hole and that, back then, life was less stressful.*" (woman, 21 years old, moderate CHD, weak SOC)

Degree of personal control	- "*I'm free to do as I like at home. I can go away quite often on trips. I can do what interests me, my own preferred sport, for instance. Consequently, I feel that I have my life under control. Also as regards what I want to study, etc.*" (man, 20 years old, complex CHD, strong SOC)<br>- "*I can't change anything. At times I wish that I was more sociable, of course. And not being so shy or making contacts faster. To step right up to people, with no fear. Like some people in my class. Just believing that it will turn out alright and not to over-prepare. But, then again, it is what it is, we can't change it any more.*" (woman, 21 years old, moderate CHD, weak SOC)

Note. CHD: congenital heart disease. SOC: sense of coherence.
